# Evaluation of a pharmacogenetic-based warfarin dosing algorithm in patients with low time in therapeutic range – study protocol for a randomized controlled trial

**DOI:** 10.1186/s12872-016-0405-1

**Published:** 2016-11-17

**Authors:** Leiliane Rodrigues Marcatto, Luciana Sacilotto, Carolina Tosin Bueno, Mirella Facin, Celia Maria Cassaro Strunz, Francisco Carlos Costa Darrieux, Maurício Ibrahim Scanavacca, Jose Eduardo Krieger, Alexandre Costa Pereira, Paulo Caleb Junior Lima Santos

**Affiliations:** 1 Laboratory of Genetics and Molecular Cardiology, Heart Institute (InCor), University of São Paulo Medical School, Av. Dr. Eneas de Carvalho Aguiar, 44 Cerqueira Cesar, São Paulo, SP CEP 05403-000 Brazil; 2 Arrhythmia Unit, Heart Institute (InCor), University of São Paulo Medical School, Av. Dr. Eneas de Carvalho Aguiar, 44 Cerqueira Cesar, São Paulo, SP CEP 05403-000 Brazil; 3 Clinical Laboratory, Heart Institute (InCor), University of São Paulo Medical School, Av. Dr. Eneas de Carvalho Aguiar, 44 Cerqueira Cesar, São Paulo, SP CEP 05403-000 Brazil

**Keywords:** Warfarin, Algorithm, Pharmacogenetic, Pharmacoeconomy, Polymorphisms

## Abstract

**Background:**

Time in therapeutic range (TTR) is a measurement of quality of warfarin therapy and lower TTR values (<50%) are associated with greater risk of thromboembolic and bleeding events. Recently, we developed a pharmacogenetic-based warfarin dosing algorithm specifically calibrated for a Brazilian patient sample. The aims of this study are: to evaluate the impact of a genetic-based algorithm, compared to traditional anticoagulation, in the time to achieve the therapeutic target and in TTR percentage; and to assess the cost-effectiveness of genotype-guided warfarin dosing in a specific cohort of patients with low TTR (<50%) from a tertiary cardiovascular hospital.

**Methods/design:**

This study is a randomized controlled trial in patients (*n* = 300) with atrial fibrillation with TTR < 50%, based on the last three INR values. At the first consultation, patients will be randomized into two groups: TA group (traditional anticoagulation) and PA group (pharmacogenetic anticoagulation). For the first group, the physician will adjust the dose according to current INR value and, for the second group, a pharmacogenetic algorithm will be used. At the second, third, fourth and fifth consultations (with an interval of 7 days each) INR will be measured and, if necessary, the dose will be adjusted based on guidelines. Afterwards, patients who are INR stable will begin measuring their INR in 30 day intervals; if the patient’s INR is not stable, the patient will return in 7 days for a new measurement of the INR. Outcomes measures will include the time to achieve the therapeutic target and the percentage of TTR at 4 and 12 weeks. In addition, as a secondary end-point, pharmacoeconomic analysis will be carried out. Ethical approval was granted by the Ethics Committee for Medical Research on Human Beings of the Clinical Hospital of the University of São Paulo Medical School.

**Discussion:**

This randomized study will include patients with low TTR and it will evaluate whether a population-specific genetic algorithm might be more effective than traditional anticoagulation for a selected group of poorly anticoagulated patients.

**Trial registration:**

ClinicalTrials.gov, NCT02592980. Registered on 29 October 2015.

## Background

Warfarin is a vitamin K antagonist and is the most widely prescribed oral anticoagulant agent worldwide [1]. It is used to prevent morbidity and mortality due to thromboembolism and, for this reason, it is very important to achieve an optimal anticoagulant therapy [2]. Furthermore, warfarin has a narrow therapeutic range and, consequently, patients usually have difficultly achieving and maintaining the therapeutic target [3–5].

Time in therapeutic range (TTR) is a measure of the quality of warfarin therapy, i.e., the percentage of time a patient’s INR (international normalized ratio) is within the desired treatment target. Lower TTR values (<50%) are associated with greater risk of thromboembolic and bleeding events; while higher TTR values (>65%) are associated with therapeutic benefits [6, 7]. Some studies identified factors that can affect TTR, such as the use of age, gender, race, and concomitant medications for predicting one’s ideal dosage [8–15]. In this scenario, some studies have assessed the impact of pharmacogenetic algorithms to guide the beginning of treatment. Pirmohamed et. al followed-up, for 12 weeks, patients who were recruited in the United Kingdom and Sweden. They used a slightly modified version of the International Warfarin Pharmacogenetics Consortium algorithm for predicting maintenance doses. They identified that the mean percentage of TTR was higher in the genotype-guided group (67.4%) compared with the control group (60.3%, *p* < 0.001) [16]. Verhoef et al studied patients taking acenocoumarol or phenprocoumon recruited in the Netherlands and Greece. They used an algorithm developed by EU-PACT group for phenprocoumon and acenocoumarol drugs [17]. They identified that genotype-guided dosing did not improve the TTR during the first 12 weeks after the initiation of therapy (61.6 and 60.2%, *p* = 0.52). However, they identified different TTR during the first 4 weeks after the initiation of treatment comparing pharmacogenetic and control groups (52.8 and 47.5%, *p* = 0.02) [18]. Kimmel et al, studied patients from 18 clinical centers in the United States. They used an algorithm developed by the COAG group [19]. The authors concluded that genotype-guided dosing of warfarin did not improve anticoagulation control during the first 4 weeks of therapy [20].

Recently, we developed a pharmacogenetic-based warfarin dosing algorithm specifically tailored for Brazilian individuals, a highly admixed population. It was shown to be more accurate than internationally developed algorithms for individuals from the Brazilian population [21]. Likewise, some other studies have shown that pharmacogenetic algorithms could be more accurate when developed and applied on specific populations [22–24].

Furthermore, studies showed the ability to improve TTR, compared with traditional anticoagulation, using strategies such as educational intervention, point-of-care testing, and the use of family practice clinics [25–27]. In this context, there is no data in the literature on the specific testing of a pharmacogenetic algorithm in patients with low TTR (<50%). In addition, cost-effectiveness evaluations for this approach are scarce and a positive finding for a pharmacogenetic approach could be very useful in a program of individualized anticoagulation therapy [28–30].

Thus, the aims of this study are: to evaluate the impact of a genetic-based, population-specific, algorithm, compared to traditional anticoagulation, in the time to achieve the therapeutic target and in the percentage of TTR at 4 and 12 weeks; and to assess the cost-effectiveness of genotype-guided warfarin dosing in a specific cohort of patients with low TTR (<50%) from a tertiary cardiovascular hospital.

## Methods

### Trial design

We will recruit 300 patients with low TTR (<50%) from the Heart Institute- Clinical Hospital-University of São Paulo Medical School (InCor- HCFMUSP).

The study protocol was approved by the Ethics Committee for Medical Research on Human Beings of the Clinical Hospital of the University of São Paulo Medical School (SDC 4033/14/013). Signed informed consent will be obtained from all participants.

**Fig. 1:**
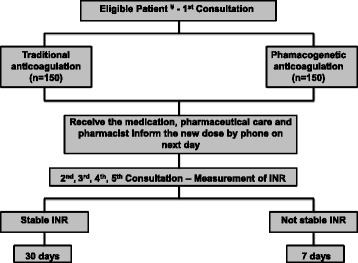
Flowchart of the study design

Figure 1 shows the study design. Physicians will select the eligible patients according to the described criteria. At the first consultation, the pharmacist will explain the study and, if the patient accepts to participate, he or she will then sign the term. Patients will be randomized into two groups: TA group (traditional anticoagulation) and PA group (pharmacogenetic anticoagulation). Randomization will be considered protocol starting date. Pharmacists will apply questionnaires and collect blood samples for DNA extraction and measure creatinine, AST and ALT.

For the TA group, the physician will adjust the dose according to the current INR value based on current guidelines [31, 32]. For the PA group, the dose will be prescribed based on data from each patient applied in a pharmacogenetic algorithm. In some cases, used algorithm may provide a counter-intuitive dose, i.e., a dose that is not adequate for adjusting the current patient’ INR (for example, a higher dose for a patient that already has a high INR). In these cases, the physician will adjust the dose following clinical criteria based on published guidelines and these patients will be kept on PA group [3, 33]. Patients of both groups will receive the medication and receive the pharmaceutical care. On the day after randomization, the pharmacist will inform, by phone, the new weekly dose prescribed for patients of both groups.

At the second (7 days), third (14 days), fourth (21 days), and fifth (28 days) consultations (with an interval of 7 days each), warfarin dosage will be adjusted, if necessary, based on current guidelines [31, 32].

After the fifth consultation, if the patient’s INR is within therapeutic target, the next measurement will be made after 30 days. If the patient’s INR is not in the therapeutic target, the warfarin dose adjustment will be made and the next INR measurement will be made after 7 days. The main outcomes will be the time to achieve the therapeutic target and the percentage of TTR at 4 and 12 weeks. We will measure the INR of all patients at the 12th week, independently of the value of INR before of this endpoint.

### Participants

Only patients with atrial fibrillation, above 18 years, and with TTR <50% based on the last three values of INR will be included in this study. Patients will be excluded if they have reached a stable dose of warfarin, liver dysfunction, alcoholism, use of another anticoagulant, use of chemotherapy, if they changed the dose of amiodarone 1 week before or if they do not meet the inclusion criteria.

### Interventions

For the TA group, the physician will adjust the dose according to the current INR value based on current guidelines [31, 32] and using an EP mobile tool for dividing the weekly dose in the days with the availability of tablets [34].

Patients with INR values <1.8 or >3.2 will undergo dose adjustment. For patients with an INR value from 1.8 to <2.0 or from >3.0 to 3.2, warfarin dose will be maintained and INR tests will continue to be made every 7 days. Then, if the patient continues to demonstrate values from 1.8 to <2.0 or from >3.0 to 3.2, warfarin dose will be changed. At each patient consultation, the pharmacist will check the drug adherence counting pills and adverse events using patient self-reporting information. The average weekly warfarin dose will be changed according to patient’s INR value: ≤1.5, increase by 20%; >1.5–<2.0, increase by 5%; >3.0–3.5, decrease by 5%; >3.5–<6.0, hold 1 dose, decrease by 15%; ≥6.0, hold warfarin and consider vitamin K based on guidelines [31, 32].

For the PA group, the dose will be prescribed based on data from each patient applied in a pharmacogenetic algorithm for the first consultation, after will adjust the dose based on guidelines and using the EP mobile [21].

### Outcomes

We will use the time to achieve the therapeutic target as primary outcomes measures and the TTR percentages as second outcome measures. We will consider that achieve the therapeutic target is three INR’s values within the therapeutic target. We will calculate the TTR in 4 and 12 weeks of follow-up.

### Sample size

With a sample size of 150 patients for each arm separately, the study will have a power of 80% to observe a difference of 8% between TTR means of the TA and PA groups, using sigma of 25% and alpha of 0.05.

### Randomization

Patients will be randomized into two groups: TA group (traditional anticoagulation) and PA group (pharmacogenetic anticoagulation). The randomization method will be by original generator, which allowed for only one block size. We will use the randomization.com to perform the randomization automatically [35]. We will add two arms with 150 subjects each. Afterward, we will include the list of randomization on the REDCap. This web application choose automatically the arm according to the list of randomization in a way that the information can not be change.

### Blinding

In this study, patients will be blinded and will not know their genetic results. The study team will not be blinded.

### Laboratorial measures and questionnaires

Oral anticoagulant therapy will be assessed by the prothrombin time (PT) in an automated coagulometric method. Venous blood samples will be collected in tubes containing sodium citrate 3.8%. INR calculation will be obtained by the ratio PT of the patient/normal PT controls, elevated to the international sensitivity index. Besides current INR, past INR values will be checked in electronic medical records. TTR will be calculated by the Rosendaal method [36], which uses linear interpolation to assign an INR value to each day between successive observed INR values. Peripheral venous blood will be collected into a BD Vacutainer System® without anticoagulant for measurements of serum creatinine, alanine aminotransferase (ALT), and aspartate aminotransferase (AST) by colorimetric and enzymatic assays.

Physicians and pharmacists will also complete a questionnaire for each patient. These will include the following variables: name, age, sex, body mass index, smoking, weekly intake of alcoholic beverages, weekly intake of dark green vegetables, comorbidities, results of last INR values, adverse events, and other drug use. In addition, the physician will evaluate the patients with the CHADS_2_VASC_2_ and HAS-BLED scores [37–39]. The CHADS_2_VASC_2_ score estimates stroke risk in patients with atrial fibrillation. The variables are congestive heart failure, hypertension, age ≥ 75, diabetes mellitus, a history of stroke or transient ischemic attack symptoms and vascular disease history [37, 38]. The HAS-BLED score estimates the risk of major bleeding for atrial fibrillation patients on anticoagulation care. “HAS-BLED” is an acronym for Hypertension (uncontrolled, systolic blood pressure >160 mmHg), Abnormal liver (cirrhosis or bilirubin >2x normal or AST/ALT/alkaline phosphatase >3x normal) and renal function (dialysis, transplant, creatinine > 2.6 mg/dL), Stroke history, Bleeding predisposition or Prior Major Bleeding, Labile INRs (unstable/high INRs or TTR < 60%), Elderly (age ≥ 65), Drugs (antiplatelet agents, nonsteroidal anti-inflammatory) or alcohol usage (≥8 drinks/week) [39]. Each patient will have an individual record, which remains with the group of pharmacists and physicians. This record will provide information on current warfarin dose (mg/week), daily dose, and INR values. Furthermore, each patient will have a card to register the warfarin daily dose.

The information gathered in the study will be managed using REDCap (Research Electronic Data Capture) tools hosted at the Clinical Hospital from the University of São Paulo Medical School. REDCap is a secure, web-based application designed to support data capture for research studies [40].

### Genotyping and predicted metabolic phenotypes

We will collect blood samples using the BD Vacutainer System® containing K_3_EDTA (Becton Dickinson, USA). We will use the QIAamp DNA Blood Kit (QIAGEN, Hilden, Germany) to extract genomic DNA from peripheral blood leukocytes. Genotyping of CYP2C9*2 (c.430C > T, rs1799853), CYP2C9*3 (c.1075A > C, rs1057910), and *VKORC1* 3673 (g.1639G > A, rs9923231) polymorphisms will be detected by real time PCR assays using Taqman probes (Applied Biosystems, CA, USA). We will use positive and negative reference samples to test along with the unknown samples in each run. TaqMan probe assay IDs: CYP2C9*2: C__25625805_10, CYP2C9*3: C___27104892_10, VKORC1 3673: C__30403261_20.

Patients will be divided into three distinct predicted phenotypes: extensive metabolizer (EM: wild-type genotypes for the *CYP2C9* polymorphisms - *1/*1), intermediate metabolizer (IM: heterozygous genotypes for the loss-of-function *CYP2C9* polymorphisms - *1/*2 or *1/*3) and poor metabolizer (PM: polymorphic homozygous or compound heterozygous genotypes for the loss-of-function *CYP2C9* polymorphisms - *2/*2 or *3/*3 or *2/*3) [41, 42].

### Pharmacogenetic algorithm

The pharmacogenetic algorithm used in this clinical study was developed and validated by our group using two independent patient cohorts. It includes the variables age, sex, weight, height, self-declared race, use of amiodarone, use of enzyme inducers, *VKORC1* genotypes, and predicted phenotypes according to *CYP2C9* polymorphisms [21].

### Pharmacoeconomic analysis

In these analyses we will evaluate the cost-effectiveness of both groups (TA and PA) calculating the costs of the genetic test, INR tests, and physician visits; and the effectiveness of the anticoagulation. We will also estimate costs that will be used to calculate the QALYs (quality-adjusted life year). These results that are used in QALYs are major bleeding, major thromboembolic events, and death. Major bleeding includes any episode of bleeding that requires hospitalization and thromboembolic events include embolic stroke, systemic embolism, ischemic attacks, deep venous thrombosis and pulmonary embolism.

### Statistical analysis

Statistical analyses will be carried out using SPSS software (v. 16.0, IBM, New York, NY) and the level of significance set at p ≤ 0.05. Pharmacoeconomic data will be evaluated using the TreeAge software (TreeAge, Inc., Williamstown, MA). Chi-square tests will be performed for comparative analysis of the categorical variables (such as adverse events, and evaluation of percentage of INR within therapeutic range) according to the polymorphisms or with the two approaches that will be made (TA and PA). Students’ *t*-tests or Mann-Whitney tests will be used for comparing TTR means and the time to achieve the therapeutic range according to the two groups. In addition, multivariate linear regression analysis will be used to identify variables associated with the TTR mean and with the time necessary to achieve the therapeutic range. A new randomization will not be performed for substitution of patients with missing data.

## Discussion

This randomized study will include patients with low TTR from a cardiovascular tertiary hospital and it was designed to evaluate whether a population-specific genetic algorithm might be more effective than traditional anticoagulation for a selected group of poorly anticoagulated patients.

Previous clinical trials tested genetic-based algorithms in patients beginning warfarin therapy and found contrasting results [43]. However, no study to date has focused on this specific group of difficult to anticoagulate patients.

Our study has some potential limitations. First, although we will check the adverse effects during the study, we will not be able to analyze them with adequate statistical power. However, since these events may severely affect cost-effectiveness analysis they are contemplated in the study design. Second, we will use an algorithm modeled from a Brazilian cohort of patients and it will be applied in a specific group of patients of a tertiary referral hospital. Thus, applicability of our findings will need of external validity.

## Abbreviations

ALT: Alanine aminotransferase
AST: Aspartate aminotransferase
CAPES: Coordenação de aperfeiçoamento de pessoal de nível superior
CNPQ: Conselho nacional de desenvolvimento científico e tecnológico
EM: Extensive metabolizer
FAPESP: Fundação de amparo à pesquisa do estado de São Paulo
IM: Intermediate metabolizer
INR: International normalized ratio
PA: Pharmacogenetic anticoagulation
PM: Poor metabolizer
PT: Prothrombin time
QALYs: Quality-adjusted life year
REDCap: Research electronic data capture
TA: Tradicional anticoagulation
TTR: Time in the therapeutic range
